# Therapeutic Management of Hypothenar Hammer Syndrome Causing Ulnar Nerve Entrapment

**DOI:** 10.1155/2010/343820

**Published:** 2010-06-07

**Authors:** Emanuele Cigna, Anna Maria Spagnoli, Mauro Tarallo, Liliana De Santo, Giampaolo Monacelli, Nicolò Scuderi

**Affiliations:** Department of Plastic Surgery, “Sapienza” University of Rome, 00185 Rome, Italy

## Abstract

*Introduction*. The hypothenar hammer syndrome is a rare traumatic vascular disease of the hand. *Method and Materials*. We report the case of a 43-years-old man with a painful tumefaction of the left hypothenar region. The ulnar artery appeared thrombosed clinically and radiologically. The patient underwent surgery to resolve the ulnar nerve compression and revascularise the artery. *Results*. The symptoms disappeared immediately after surgery. The arterial flow was reestablished. Postoperatively on day 20, a new thrombosis of the ulnar artery occurred. *Conclusion*. Hypothenar hammer syndrome is caused by repetitive trauma to the heel of the hand. The alterations of the vessel due to its chronic inflammation caused an acute compression of the ulnar nerve at the Guyon's canal and, in our case, do not allow a permanent revascularisation of the ulnar artery.

## 1. Introduction

The hypothenar hammer syndrome (HHS) is a rare clinical condition, first described by Von Rosen in 1934 [1] and named by Conn in 1970 [2], which may cause pain, a mass in the hand or finger's ischemia. The syndrome is caused by blunt traumas to the heel of the hand that may damage the ulnar artery at the level of the hypothenar eminence, resulting in aneurysm or occlusion of the vessel [1–5].

The therapeutic approach of this syndrome is strictly correlated to its symptoms starting from a simple oral antiplatelet aggregation therapy to a surgical excision of the affected vessel and its reconstruction with a venous bypass [5–7]. 

We report a case in which the posttraumatic chronic inflammation of the thrombosed ulnar artery caused an acute ulnar nerve compression at the Guyon's canal. After surgery the patient became asymptomatic, but a new thrombosis occurred.

## 2. Case Report

A 43-year-old man, manual worker, heavy smoker (1-2 packs/day), came to the emergency department with a 2-day history of an erithematous painful tumefaction of the left hypothenar eminence that in the last 12 hours became very painful particularly involving the ring and little fingers. 

Physical examination showed unilateral clinical manifestations involving the dominant hand. Palpable pulses of the left radial and ulnar arteries. 

The patient was a middle-aged man whose occupational activities required the excessive and harmful use of the hypothenar region.

The patient was a bus driver, which was exposed to vibration. A traumatic chronic inflammation of ulnar artery occurred, resulting in arterial injury.

The patient reported tingling sensation, parestesia, dysesthesia, pain, and reduction of the sensitivity of the ring and little finger (territory of innervation of the ulnar nerve).

This is instead normal sensitivity on the back of the hand and fingers.

**Figure 1:**
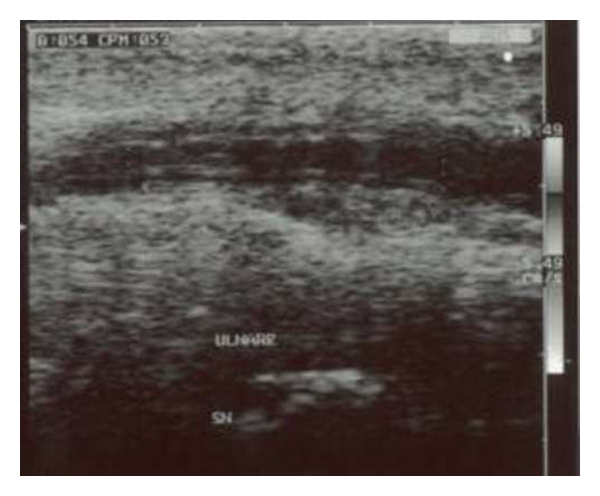
Longitudinal sonographic image of a left ulnar artery in a patient with hypothenar hammer syndrome. The artery appeared thrombosed and thicker with a “triple ring aspect” of its wall.

Allen's test indicates a radial dominance for the blood supply of the hand. Tinel's sign showed an ulnar nerve sufferance at the Guyon's canal. No signs of ulnar nerve damage were observed but were present signs of nerve compression. Laboratory investigation for haematological disorders or connective tissue diseases were negative except for a thrombocytopenia (PTL 87,000x mm^3^). He was referred for doppler-sonography evaluation and MR imaging. Gray scale and doppler ultrasounds showed a thrombosed ulnar artery at the level of the hook of the hamate. Doppler-sonography evaluation showed thrombosis, oedema, and nerve compression. The artery appeared thrombosed and thicker with a “triple ring aspect” of its wall (Figure 1). MRI showed diffused oedema in the hypothenar eminence. Surgery objectives were to resolve the pain and revascularise the ulnar artery. 

Semmes-Weinstein monofilament test and elettromiography were not performed for two main reasons listed above (the patients came in emergency and there was the possibility of digital complications).

## 3. Results

All our goals were accomplished. The ulnar artery appeared thrombosed and increased on its calibre, it compressed the nerve at the Guyon's canal. The canal was released and an endoaterectomy (Fogarty catheter n°3) was performed in order to reestabilish the flow and avoid any risk of mircoclots genesis that could occlude digital arteries. The pain disappeared immediately after brachial plexus anaesthesia resolved. The patient was treated postoperatively with a low molecular weight heparin (enoxaparin 0.4 4000 UI/day × 15 days Clexane) and controlled clinically with the Allen's test and instrumentally with CW doppler sonography to evaluate the ulnar flow. However, on postoperative day 20 a doppler-sonography evaluation was performed and we assisted of a new silent thrombosis of the artery at the same level.

The patient was informed that the best treatment was surgical excision of the affected vessel and its reconstruction with a venous bypass. Patient was not feeling any pain and he refused the treatments.

## 4. Discussion

The hypothenar hammer syndrome is a rare disease caused by lesions of the ulnar artery secondary to trauma. This syndrome usually occurs following chronic, repetitive blunt trauma [1–8], but may rarely follow also a single acute episode [8]. The vulnerability of the ulnar artery at the level of the hypothenar region is probably caused by the anatomy of the Guyon's canal. Continuous compression of the vessel between external surfaces and the palmar hamate bone may cause a damage of the intima resulting in platelet aggregation and thrombus formation, with aneurismal dilatation if damage is extended also to the media layer [2]. 

Several authors report that in the hypothenar region, the unique anatomy of the Guyon canal allows the arterial injury though traumas [9–11]. In fact hook of the hamate strikes the superficial palmar branch of the ulnar artery in the Guyon space, leading to the occlusion and/or aneurysm of the ulnar artery.

This syndrome has generally been described in the dominant hand of males who, during occupational [4, 12] or recreational activities [13–20], use the heel of the hand as a hammer. 

In 2000 Ferris et al. [21] proposed that the posttraumatic alteration of the ulnar artery may appear only in people predisposed, on the basis of the low percentage of the affected people compared to all the people that has the local traumatic behaviour; he also hypothesize that one or more unknown factors predisposed the ulnar artery to damage. In our opinion HHS is a multifactorial disease. One of these factors, almost certainly the most significant, is represented by an abnormal vascular restoration named fibromuscular dysplasia [21]. This hypothesis may also be confirmed by the often bilateral nature of the HHS, first suggested by Conn, if the injuries affect both hands [2]. Traumas and fibromuscular displasia, a sort of “major risk factors” made the ulnar artery prone to the disease. Probably there are many factors of minor importance (“minor risk factors”), one of these is certainly the smoke that affects the 76% of patients with HHS [21]. 

The HHS is clinically characterized by pain and tumefaction of the hypothenar eminence. 

Frequently the thrombus in the ulnar artery causes vascular visible disorders such as whitening of the smitten fingers for the continue microembolisation into the digital arteries 19–21 that may simulate a Raynaud's fenemenon [8, 22, 23]. 

Diagnosis of HHS should be suspected clinically in cases of a history of trauma (hand workers or athletes) pain over the hypothenar area (not always present) and a pathologic Allen's test [24]. 

Definitive diagnosis can be confirmed on the basis of the instrumental vessel occlusion demonstration to show the flow alteration. CW-doppler sonography [1, 7, 25], digital angiography [5, 24, 26–28], or angiography MRI [29] have been utilised with comparable results. 

In HHS patients, angiography shows occlusion and aneurysm of the ulnar artery in the area of the Guyon space, causing compression of the ulnar nerve with possible occlusions of the digital arteries [9]. 

The possibilities of immunological or haematological disorders have to be excluded with selected laboratory investigations [29].

Various therapies have been proposed for HHS. According with the literature, the approach should be guided by clinical symptoms [6, 21, 30–34]. In case of documented finger's ischemia for thrombosis or aneurysm formation, surgical resection with end-to-end anastomosis, or venous bypass reconstruction is required. In cases without symptoms a conservative treatment with aspirin and nifedipine is usually adopted [3, 30, 35]. Good results have been reported with infusion of heparin, vasodilatators, low molecular weight dextran, local thrombolysis but also with radical approaches stellate ganglion blockades or thoracic sympathectomy [3, 36, 37]. 

**Figure 2:**
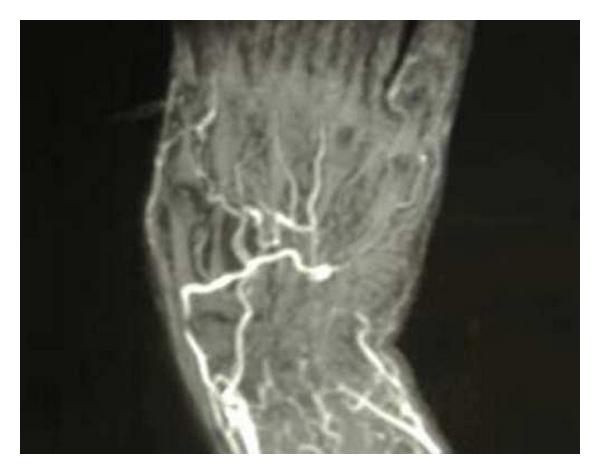
Angio MRI of the left hand showing the ulnar occlusion at the level of the Guyon's canal.

**Figure 3:**
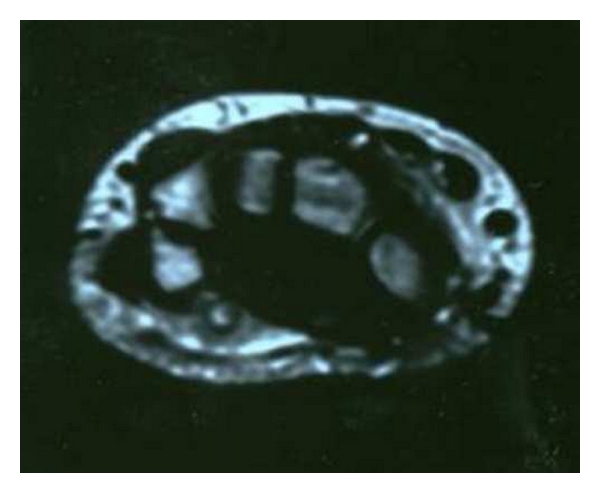
MRI showing a thrombosis of the ulnar artery, an inflammation of the ulnar nerve and surrounding tissue at the level of the Guyon's canal.

In our case patient's job required the pressing of a bulb handled level with the affected hand. The chronic inflammation and oedema of the artery, and of the soft tissues surrounding it, caused the start of symptoms for the ulnar nerve entrapment in the Guyon's canal (Figures 2 and 3). The progressively worsening pain at the left ring and little fingers forced the patient to come to our department. 

**Figure 4:**
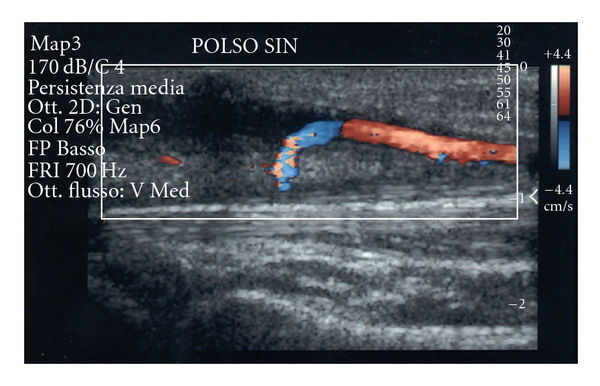
Color doppler study of the ulnar artery on postoperative day 20 showing a new thrombosis on the same site of the ulnar artery.

In this case the patient has been operated for the symptoms that were probably caused only by ulnar nerve entrapment, resolved by surgery. Doppler-sonography evaluation showed thrombosis, oedema, and nerve compression confirmed by an angio-RM. At the time of surgery the ulnar artery appeared thicker and thrombosed, the nerve was compressed but no appearing damages as a chronic cases. We decided a conservative approach with catheter thinking on a recent thrombosis of the vessel. At the time of new thrombosis, on postoperative day 20, (Figure 4) on the basis of the nonsymptomatic occlusion, we changed the treatment options versus a conservative, nonsurgical, approach with low-dose aspirin and calcium blockers (nifedipine) [4, 35]. The follow up at 20 months with angiography MRI shows the stability of the arterial flow of the hand. 

Probably the reason why we assisted of a new silent thrombosis of the artery at the same level was the endothelial damage and the vascular inflammation that was the base assumptions of the pathological condition. This thrombosis was silent because the nerve was released. When the new thrombosis occurred, patient was informed that the best treatment was surgical excision of the affected vessel and its reconstruction with a vein graft. Patient was asymptomatic and refused the treatments.

## 5. Conclusions

The rarity of the hypothenar hammer syndrome generally cause a delayed diagnosis because the syndrome may go undetected for flow compensation through radial artery and probably is underestimated in hand workers. Therapy approaches, in non-symptomatic cases, are observation and risk factor management. In our case surgery resolved the nerve entrapment causing the pain. At the time of the new thrombosis the surgical approach, in a asymptomatic patient, well managed by drug therapy, is not yet justified.
